# Integrated Genetic Screening Identifies FEN1 as a Selective Vulnerability and Temozolomide Resistance Mediator in Glioblastoma Stem Cells

**DOI:** 10.1158/2767-9764.CRC-26-0150

**Published:** 2026-10-05

**Authors:** Benjamin A. Brakel, Dillon McKenna, Anish Puri, Muhammad Vaseem Shaikh, Manoj Singh, Ali Saleh, Abdo-jose Tomajian, Nicholas Mikolajewicz, Marcello Beltrami, Maxwell Topley, Alisha Anand, Petar Miletic, Kevin R. Brown, David Tieu, William Maich, Sabra Salim, Yujin Suk, Minomi Subapanditha, Deena M.A. Gendoo, Chitra Venugopal, Jason Moffat, Sachin Katyal, Chirayu R. Chokshi, Sheila K. Singh

**Affiliations:** 1 Department of Biochemistry and Biomedical Sciences, McMaster University, Hamilton, Canada.; 2 Centre for Discovery in Cancer Research, McMaster University, Hamilton, Canada.; 3 Department of Surgery, Faculty of Health Sciences, McMaster University, Hamilton, Canada.; 4 Paul Albrechtsen Research Institute, CancerCare Manitoba, Winnipeg, Canada.; 5 Department of Pharmacology and Therapeutics, Max Rady College of Medicine, Rady Faculty of Health Sciences, University of Manitoba, Winnipeg, Canada.; 6 Program for Genetics and Genome Biology, The Hospital for Sick Children, Peter Gilgan Research and Learning Centre, Toronto, Canada.; 7 Department of Cancer and Genomic Sciences, School of Medical Sciences, College of Medicine and Health, University of Birmingham, Birmingham, United Kingdom.; 8 Donnelly Centre, University of Toronto, Toronto, Canada.; 9 Department of Molecular Genetics, University of Toronto, Toronto, Canada.; 10 Institute for Data and AI, University of Birmingham, Birmingham, United Kingdom.; 11 Institute for Biomaterials and Biomedical Engineering, University of Toronto, Toronto, Canada.; 12 School of Cancer and Pharmaceutical Sciences, Comprehensive Cancer Centre, King's College London, London, United Kingdom.

## Abstract

Glioblastoma (GBM) is an aggressive brain tumor characterized by therapy resistance and recurrence. GBM stem cells (GSC) are key drivers of tumor maintenance, therapeutic resistance, and relapse, but targeting them remains clinically elusive due to their overlap with normal neural stem cells (NSC) and a lack of actionable vulnerabilities. To identify selective vulnerabilities in GSCs, we performed genome-wide CRISPR-Cas9 loss-of-function screening across patient-derived GSC models under standard-of-care treatment conditions. We identified flap endonuclease 1 (FEN1), a key enzyme in DNA replication and base excision repair, as an essential gene for GSC survival, with enhanced dependency in the context of temozolomide (TMZ) treatment. Genetic knockdown of *FEN1* impaired GSC proliferation and self-renewal and extended survival in a patient-derived xenograft model. Pharmacologic inhibition of FEN1 using a small-molecule inhibitor revealed selective cytotoxicity in highly aggressive and recurrent GBM models while sparing NSCs. Notably, FEN1 inhibition synergized with TMZ to induce DNA double-strand breaks and potentiate cell death only in a subset of GSCs sensitive to FEN1 inhibition. Mechanistically, single-cell transcriptomics revealed that *FEN1* expression correlates with programs linked to proliferation, stemness, and DNA damage repair, underscoring its role in maintaining the treatment-refractory phenotype. Our findings identify FEN1 as a selective vulnerability in aggressive, proliferative GSCs. FEN1 inhibition not only impairs GSC viability but also restores sensitivity to TMZ in treatment-resistant models, offering a strategy for salvage therapy in recurrent GBM. These results support the development of FEN1-targeted therapies and lay the foundation for a biomarker-guided approach to overcoming chemoresistance in GBM.

**Significance:**

GBM is a lethal brain tumor driven by therapy-resistant GSCs. We identified FEN1 as a selective vulnerability of GSCs that promotes TMZ resistance. FEN1 inhibition disrupts GSC self-renewal, restores TMZ sensitivity, and spares healthy NSCs, establishing FEN1 as a precision neuro-oncology target.

## Introduction

Glioblastoma (GBM) is the most common and aggressive primary brain tumor in adults, marked by rapid progression, therapy resistance, and dismal outcomes. Despite multimodal treatment, including surgical resection, radiotherapy (RT), and temozolomide (TMZ) chemotherapy, the median survival remains less than 15 months, with inevitable tumor relapse (1). Therapy resistance is largely attributable to the presence of GBM stem cells (GSC), a treatment-resistant and highly tumorigenic subpopulation that drives tumor growth, resistance to genotoxic therapies, and recurrence. GSCs exhibit enhanced DNA repair capacity, stem-like properties, and dynamic adaptability, rendering them inherently resistant to standard-of-care (SoC) treatments (2–5). However, the definition of GSCs remains an area of active investigation and debate, as these populations are functionally and phenotypically heterogeneous and may exist along a dynamic spectrum of cellular states rather than as discrete entities (6, 7). Despite this, a substantial body of evidence supports the presence of stem-like, therapy-resistant subpopulations that contribute to tumor propagation and recurrence in GBM (2, 3, 7).

Although genomic and transcriptomic profiling have offered insights into the complex molecular landscape of GBM, these static approaches fail to capture the functional drivers of GSC survival and therapy resistance. Among established mechanisms of resistance to TMZ, O6-methylguanine-DNA methyltransferase (MGMT) promoter methylation status remains one of the most clinically relevant biomarkers, with promoter methylation associated with improved response to TMZ due to reduced DNA repair capacity. Conversely, MGMT expression confers resistance by reversing TMZ-induced DNA damage, highlighting the importance of DNA repair pathways in mediating the therapeutic response. However, this and other established mechanisms do not fully account for the complexity of therapeutic resistance in GBM, and the functional drivers underlying treatment failure remain incompletely understood.

The advent of CRISPR-Cas9 technology has revolutionized functional genomics, enabling genome-wide screens to uncover essential genes in an unbiased manner (8). However, few efforts have focused on understanding the functional drivers of therapy resistance in GBM, particularly in the context of SoC chemoradiotherapy, leaving a gap in understanding how genetic dependencies evolve in response to standard treatments. In this study, we conducted genome-wide CRISPR knockout screens in patient-derived GSCs to systematically identify genetic vulnerabilities associated with GSC survival and resistance to therapy. By integrating functional genomic data across treatment conditions, we explored conditional genetic interactions of essential genes to ultimately identify dependencies that both sustain GSC viability and mediate resistance to SoC therapies. These efforts identified flap endonuclease 1 (FEN1) as a key driver of GSC survival, with a heightened dependency under TMZ treatment. Further analysis revealed that FEN1 inhibition selectively targets highly aggressive, stem-like GBM cells, revealing a therapeutic vulnerability that not only disrupts GBM growth but also enhances TMZ efficacy in treatment-resistant tumors.

These findings help illuminate the genetic underpinnings of GSC survival and therapy resistance, offering a new framework for targeting functional vulnerabilities in GBM. By identifying FEN1 as a cancer-selective and therapy-sensitizing target, this work provides a basis for developing precision therapies aimed at overcoming resistance and improving outcomes for patients with this devastating disease.

## Materials and Methods

Our research complies with ethical regulations according to the Hamilton Health Sciences and McMaster Health Sciences Research Ethics Boards. Studies were conducted in accordance with the International Ethical Guidelines for Biomedical Research Involving Human Subjects (CIOMS), and animal studies were approved by the McMaster Animal Research Ethics Board.

### Derivation and culture of patient-derived GSC models

Primary GBM specimens and whole fetal brain samples were collected from patients and families who gave written informed consent, following approval from the Hamilton Health Sciences and McMaster Health Sciences Research Ethics Board. After an initial PBS wash, specimens underwent mechanical dissociation, followed by enzymatic dissociation in PBS containing 0.013 mg/mL Liberase Thermolysin Research Grade (MilliporeSigma, cat. #5401020001) at 37°C for 15 minutes. The dissociated cells were filtered through a 70 μm cell strainer (MilliporeSigma, cat. #CLS431751-50EA), centrifuged, and subjected to red blood cell lysis using a 0.8% ammonium chloride solution (STEMCELL Technologies, cat. #07850).

Tumor cells and fetal human neural stem cells (NSC) were cultured in the NeuroCult NS-A Proliferation Kit (Human; STEMCELL Technologies, cat. #05751), supplemented with 20 ng/mL EGF (STEMCELL Technologies, cat. #78006), 10 ng/mL FGF (STEMCELL Technologies, cat. #78003.2), 0.002% heparin (w/v; STEMCELL Technologies, cat. #07980), and 1× antibiotic/antimycotic solution (Wisent Bioproducts, cat. #450-115-EL). During the initial 2 weeks of culture, cells were treated with 1× MycoZap Prophylactic (Lonza, cat. #VZA-2031) to prevent potential mycoplasma contamination.

Cells were maintained on tissue culture–treated dishes, cell-repellent dishes (Greiner Bio-One, cat. #628979), or tissue culture–treated dishes coated with poly-L-ornithine (MilliporeSigma, cat. #P4957) and mouse laminin (Corning, cat. #354232). All cultures were kept at 37°C in a 5% CO_2_ environment. Cell authentication via short tandem repeat sequencing was performed at the initial cell line derivation and periodically as cells were passaged. Regular mycoplasma testing was performed using a MycoAlert Mycoplasma Detection Kit (Lonza) within approximately 1 year of experimental use, and if necessary, cells were treated with an additional 2-week course of 1× MycoZap. Passaging of cells was limited, and low-passage samples were used for all experiments included in the study.

### Genome-wide CRISPR-Cas9 knockout screening

For our in-house screens, the Toronto KnockOut version 3.0 (TKOv3) guide RNA (gRNA) library (Addgene, cat. #90294) was packaged into lentivirus, and the multiplicity of infection (MOI) was determined as previously reported (9). Our dropout screen (in one GBM cell line, BT935) was performed with methodology as described by our group previously (10). gRNA read counts were acquired from additional in-house screens (BT972, BT241, and BT594; ref. 10) and others (G440_L, G523_L, G729_L, G532_L, G620_L, BT67_L, G361_L, G691_L, G809_L, G549_L, G583_L, CB660, U5, HF7450, and HF6562; refs. 11–13). For our drug-treated screen (in one GBM cell line, BT935), infected and puromycin-selected cells were split into one of four treatment arms: control dimethyl sulfoxide (DMSO) treatment, sublethal IC_20–30_ RT, sublethal IC_20–30_ TMZ, or sublethal IC_20–30_ RT and TMZ. For the combined RT and TMZ arm, each plate was treated with TMZ (MilliporeSigma, cat. #T2577) 1 hour prior to RT (Faxitron RX-650). For the entire duration of the screen, treatments were administered in cycles of 5 days on and 2 days off to allow for recovery and passaging. Library preparation, Illumina sequencing of gRNAs, mapping and quantification of gRNAs, quality control analysis, and gRNA scoring were performed as previously described (9). In summary, to quantify gene fitness in each screen, the log fold change (LFC) of each gRNA was quantified by comparing read depth–normalized gRNA counts at the beginning (T0) and end (Tn) of each screen. LFC values for all gRNAs targeting a single gene were averaged, with four gRNAs targeting each gene in TKOv3 (9). Core essential gene sets (reference; refs. 8, 9, 14) and nonessential gene sets (background; refs. 8, 9) were used to assess quality control. Where indicated, LFC values for gRNAs targeting each gene were compared between a drug-treated arm and the DMSO-treated arm for each cell line by applying the DrugZ algorithm (15).

For dropout screens, LFC values of core essential genes and nonessential genes were used to apply the Bayesian Analysis of Gene Essentiality (BAGEL) algorithm (16) to determine gene fitness. A Bayes factor (BF) score of ≥5 and a false discovery rate (FDR) < 0.05 were used to identify genes essential for cell survival. GBM-enriched genetic dependencies were identified as genes essential across all GBM models, after excluding genes from core essential gene datasets and those essential in a majority of NSC lines (9, 14). For the drug-treated screen (BT935), LFC of normalized gRNA read counts was compared between control (DMSO) and treatment (RT + TMZ) arms.

### Knockout and knockdown generation

CRISPR knockout gRNA targeting *AAVS1* (5′-GGG​GCC​ACT​AGG​GAC​AGG​AT-3′), *FEN1* (5′-TCT​GAG​GAG​CGA​ATC​CGC​AG-3′), or *TP53* (5′-GCA​GTC​ACA​GCA​CAT​GAC​GG-3′) was obtained from TKOv3 (9) and cloned into the single-gRNA lentiCRISPRv2 construct (Addgene, cat. #52961). For knockdowns, hairpin oligonucleotides were constructed targeting the coding sequence of *GFP* (5′-TGA​CCC​TGA​AGT​TCA​TCT​GCA-3′) or *FEN1* (5′-GCA​GTG​ACT​ACT​GTG​AGA​GTA-3′) and cloned into the pLKO.1 construct (RRID: Addgene_10878). Constructs were validated using Sanger sequencing. Each construct was packaged into lentivirus using second-generation packaging vectors [psPAX2 (RRID: Addgene_12260) and pMD2.G (RRID: Addgene_12259) with polyethyleneimine transfection reagent], and tumor cells were infected as described above at an MOI of 1. Twenty-four hours after infection, virus-containing media were replaced with fresh medium containing puromycin (1–3 μg/mL), and cells were incubated for an additional 24 to 72 hours. Knockout assays were performed in three GBM cell lines (BT935, BT972, and BT241), and knockdown assays were performed in one GBM cell line (BT972).

### *In vitro* assays for self-renewal and proliferation

#### Cell preparation

Tumor cells or NSCs, with or without genetic knockout, were dissociated into a single-cell suspension using incubation with TrypLE Express Enzyme (Thermo Fisher Scientific, cat. #12605028) for 5 minutes at 37°C. After washing with PBS, the cell suspension was filtered through a 35 μm cell strainer and plated for assays. All assays were performed using the NeuroCult NS-A Proliferation Kit (Human), supplemented as described above.

#### Secondary sphere formation assays

Cells were plated at 200 cells/well in flat-bottom tissue culture-treated 96-well microplates. Cells were cultured in 200 μL of medium and incubated for 7 days at 37°C and 5% CO_2_. Following incubation, cell spheres were counted by microscopy. An unpaired *t* test was used to compare samples.

#### Proliferation assays

Cells were plated at 1,000 cells/well in flat-bottom tissue culture-treated 96-well microplates. Cells were cultured in 200 μL of medium and incubated for 7 days at 37°C and 5% CO_2_. Compounds, if used, were added at the desired concentrations, maintaining a constant volume of vehicle across treatments/controls. All experiments were conducted with three to five experimental replicates per sample. To quantify proliferation, 20 μL of PrestoBlue cell viability reagent was added to each well, followed by incubation for 4 hours at 37°C and 5% CO_2_. Fluorescence was measured on a FLUOstar Omega Fluorescence 566 microplate reader (BMG LABTECH; excitation 540 nm and emission 570 nm) and analyzed using Omega software. An unpaired *t* test was used to compare samples. For FEN1 inhibitor dose–response assays, cells were treated with FEN1 inhibitor or DMSO (vehicle control) once and incubated for 7 days before readout. GBM models were classified as “FEN1 inhibitor–sensitive” or “FEN1 inhibitor–resistant” based on their viability response to FEN1 inhibition, with sensitive lines demonstrating a marked reduction in cell viability at low micromolar concentrations of inhibitor, and resistant lines showing minimal or no reduction under the same conditions. For TMZ dose–response assays, cells were treated with TMZ or DMSO for 5 consecutive days at the same dose, and assay readout was 7 days after plating. Paclitaxel (PTX) assays were done in a similar fashion, after culturing cells with 40 nmol/L PTX or control DMSO for 6 days prior to plating the assay, with readout occurring 7 days after plating the assay.

### *In vivo* xenograft model

Tumor cells from one GBM cell line (BT972) were orthotopically injected into immunocompromised mice as previously described (2). In summary, NOD-SCID mice aged 6 to 12 weeks were anesthetized with isoflurane (5% for induction, 2.5% for maintenance). A total of 100,000 tumor cells from cultured cell lines were suspended in 10 μL of PBS and administered into the right frontal lobe of each mouse. Tumor presence was verified via MRI. Mice were euthanized upon reaching a humane endpoint or experiencing a 20% weight loss. Survival analysis was performed in R using the survfit() and surv() functions from the “survival” package (version 3.2-3) and visualized with the “survminer” package (version 0.4.8).

### *MGMT* promoter methylation

To determine *MGMT* promoter methylation status across cell cultures, methylation-specific PCR was used. For each cell culture, DNA was extracted using the QIAGEN QIAamp DNA Mini Kit (cat. #56304). A total of 200 ng of DNA then underwent bisulfite conversion using the QIAGEN EpiTect Bisulfite Kit (cat. #59104). Bisulfite-converted DNA was subjected to a two-stage nested PCR as previously described (17). Stage 1 amplification primers recognize bisulfite-converted DNA but do not amplify methylation-specific promoter regions. The primers used for stage 1 were as follows: MGMT-forward, 5′-GGA​TAT​GTT​GGG​ATA​GTT-3′, and MGMT-reverse, 5′-CCA​AAA​ACC​CCA​AAC​CC-3′. Hot Start Taq DNA Polymerase (New England Biolabs, cat. #M0495S) was used in a 50 μL reaction, and the master mix was prepared according to the manufacturer’s specifications. Stage 1 thermocycling conditions were as follows: initial denaturation at 95°C for 5 minutes, followed by 40 cycles of denaturation at 95°C for 30 seconds, annealing at 52°C for 30 seconds, and extension at 72°C for 30 seconds, with a final extension at 72°C for 5 minutes. The stage 1 PCR product was diluted 10-fold and used as the template for the stage 2 PCR. Primers used to amplify methylated and unmethylated promoter regions were as follows: methylated forward primer, 5′-TTT​CGA​CGT​TCG​TAG​GTT​TTC​GC-3′; methylated reverse primer, 5′-GCA​CTC​TTC​CGA​AAA​CGA​AAC​G-3′; unmethylated forward primer, 5′-TTT​GTG​TTT​TGA​TGT​TTG​TAG​GTT​TTT​GT-3′; and unmethylated reverse primer, 5′-AAC​TCC​ACA​CTC​TTC​CAA​AAA​CAA​AAC​A-3′. Commercially available globally methylated DNA (Sigma-Aldrich, cat. #S7821-M) and unmethylated DNA (Sigma-Aldrich, cat. #S7822-M) were used as positive controls, whereas a water blank was used as a negative control. Stage 2 PCR conditions were as follows: initial denaturation at 95°C for 5 minutes, followed by 40 cycles of denaturation at 95°C for 50 seconds, annealing at 59°C for 50 seconds, and extension at 72°C for 50 seconds, with a final extension at 72°C for 5 minutes. PCR products were run on a 2% agarose gel at 120 V to separate the methylated amplicon (81 bp) from the unmethylated amplicon (93 bp).

### RNA sequencing

RNA sequencing (RNA-seq) data were used to quantify the expression of mismatch repair (MMR) genes and to identify putative coding variants across the six GBM cell lines used in the study, as previously described (10).

### Synergy assays

Tumor cells from six GBM cell lines were plated and treated as described for proliferation assays, with increasing TMZ and FEN1 inhibitor concentrations while keeping a constant DMSO (vehicle) concentration. Expected inhibition was calculated using Bliss additivity, and synergy was calculated using Bliss excess, as previously described (18), using SynergyFinder (v2.0).

### Immunoblotting

For Western blotting, cell pellets were thawed and lysed immediately. Protein extracts were prepared using lysis buffer [50 mmol/L Tris-HCl, 200 mmol/L NaCl, 0.2% NP-40, 1% Tween 20 (v/v)] supplemented with 1 mmol/L NaF, 1 mmol/L sodium vanadate, 50 mmol/L β-glycerophosphate, 2 mmol/L phenylmethylsulfonyl fluoride, and a complete protease inhibitor (Roche). Lysates were then centrifuged at 13,000 rpm for 10 minutes, and the supernatant was collected. Protein concentration was determined by Bradford assay (Bio-Rad); proteins (20 μg per lane) were separated through a Bolt 4% to 12% (w/v) Bis-Tris Plus gel (Life Technologies) and transferred onto a nitrocellulose membrane (Bio-Rad). Blots were sequentially immunostained with the antibodies indicated in Supplementary Table S1. For secondary antibodies, blots were subsequently incubated with either horseradish peroxidase–conjugated goat anti-mouse (Bio-Rad, cat. #1706516, RRID: AB_2921252, 1:2,000) or goat anti-rabbit (Bio-Rad, cat. #1706515, RRID: AB_11125142, 1:2,000) and detected using Clarity chemiluminescence reagent (Bio-Rad) and imaged using an ImageQuant LAS 500 automated chemiluminescence imager (GE HealthCare). Densitometry analysis of individual protein band signals was performed via ImageJ.

### Comet assay

Tumor cells from one primary (BT594) and one recurrent (BT972) GBM cell line were dissociated into a single-cell suspension using 5 μL Liberase Blendenzyme (0.2 Wünsch unit/mL, Sigma, cat. #5401119001) in 1 mL PBS for 5 minutes at 37°C. Cells were plated at a density of 3,000 to 5,000 cells/well in 100 μL of culture media in a 96-well Ultra-Low Attachment Surface plate (Costar, Corning, cat. #3474). After 48 hours of treatment with TMZ (0–200 μmol/L) and/or FEN1 inhibitor (30 μmol/L), cells were collected, embedded in low-melting agarose, and transferred onto 96-well comet slides. The slides underwent lysis to remove membranes, an alkaline buffer to unwind the DNA, and then electrophoresis at 4°C for 40 minutes at 21 V, after which the slides were washed, dried, and stained with SYBR Green. DNA damage was visualized using a Cytation 5 cell imaging reader and analyzed with Gen5 software, using comet tail moment as an indicator of DNA damage.

### Ki67 immunofluorescence

Tumor cells from six GBM cell lines were cultured in cell culture chamber slides until they reached approximately 70% to 80% confluence. The cells were then fixed with 10% formalin for 20 minutes at room temperature, followed by three washes with PBS. Permeabilization was performed using 0.1% Triton X-100 in PBS for 15 minutes at room temperature. After an additional three washes with PBS, cells were blocked with 5% bovine serum albumin (BSA) in PBS for 1 hour at room temperature to prevent nonspecific binding.

Cells were incubated overnight at 4°C with a primary antibody against Ki67 (rabbit anti-Ki67 antibody, Thermo Fisher Scientific, cat. #RM-9106-S0, RRID: AB_2341197) diluted 1:1,000 in 1% BSA/TBS. The following day, cells were washed 3 times with PBS and incubated with a fluorophore-conjugated secondary antibody (goat anti-rabbit IgG (H + L) Alexa Fluor 488, Thermo Fisher Scientific, cat. #A-11008, RRID: AB_143165) diluted 1:500 in 0.1% BSA/TBS for 2 hours at room temperature in the dark. After washing 3 times with PBS, nuclei were counterstained with 4′,6-diamidino-2-phenylindole (DAPI) for 5 minutes. Coverslips were then mounted and sealed.

Fluorescent images were acquired using a fluorescence microscope with 20× magnification. Image processing and quantification were performed using ImageJ.

### Single-cell RNA-seq analysis

Single-cell RNA-seq (scRNA-seq) data from Abdelfattah and colleagues (19) were obtained from Gene Expression Omnibus (GEO; accession number GSE182109), and those from Wang and colleagues (20) were obtained from GEO (accession number GSE131928). scRNA-seq datasets were normalized using the *Seurat* (v4.2.1) workflow (21). In brief, count matrices for each tumor were loaded into separate Seurat objects and normalized using the NormalizeData function (…) with default parameters. Normalized values were used for gene expression and correlation analyses.

The curated list of gene sets that were used for gene program scoring included HALLMARK (22), Gavish hallmark cancer programs (23), Greenwald GBM subtypes (24), Neftel GBM subtypes (5), Verhaak GBM subtypes (25), CancerSEA (26), and IVY spatial signatures (27), along with select stemness-associated gene sets, including StemSig (28), iPSC (29), Nanog/Sox2/ES1 (30), and StemCell (31). We also calculated additional stemness indices using the Correlation of Connectome and Transcriptome (32) and StemSC (33). Gene set activities were calculated using the AddModuleScore (…) function in Seurat (21), which corrects for baseline expression to account for technical and biological variability.

Spearman correlations between each gene program and *FEN1* expression were calculated within each patient tumor and averaged across all tumors. Significance for correlations and pairwise expression comparisons was determined using Wilcoxon tests followed by *post hoc* Benjamini–Hochberg corrections.

To evaluate *FEN1* gene-level associations, the normalized codependency index (nCDI) was calculated at the tumor level between *FEN1* and each gene method using the FindCDIMarkers (…, features.x = “FEN1”) function with default parameters from the *scMiko* R package (v1.0.0; ref. 34). CDI scores across all tumors were then averaged and rank-ordered to highlight the top *FEN1*-associated genes. FEN1-associated genes were defined as those that satisfied the following inter- and intratumoral significance criteria: (i) The average of all tumor-level nCDI scores was significantly greater than zero, at a 5% FDR, and (ii) tumor-level nCDI was significant at a 5% FDR in at least 50% of tumor samples.

### Statistical analysis

All experimental data are accompanied by the number of experimental replicates in the figure legends or text. Unless stated otherwise, statistical significance was assessed using an unpaired Student *t* test, with *, *P* < 0.05; **, *P* < 0.01; ***, *P* < 0.001; and ****, *P* < 0.0001. Statistical analyses were performed using R.

## Results

### Genome-scale identification of genetic dependencies

**Figure 1.**
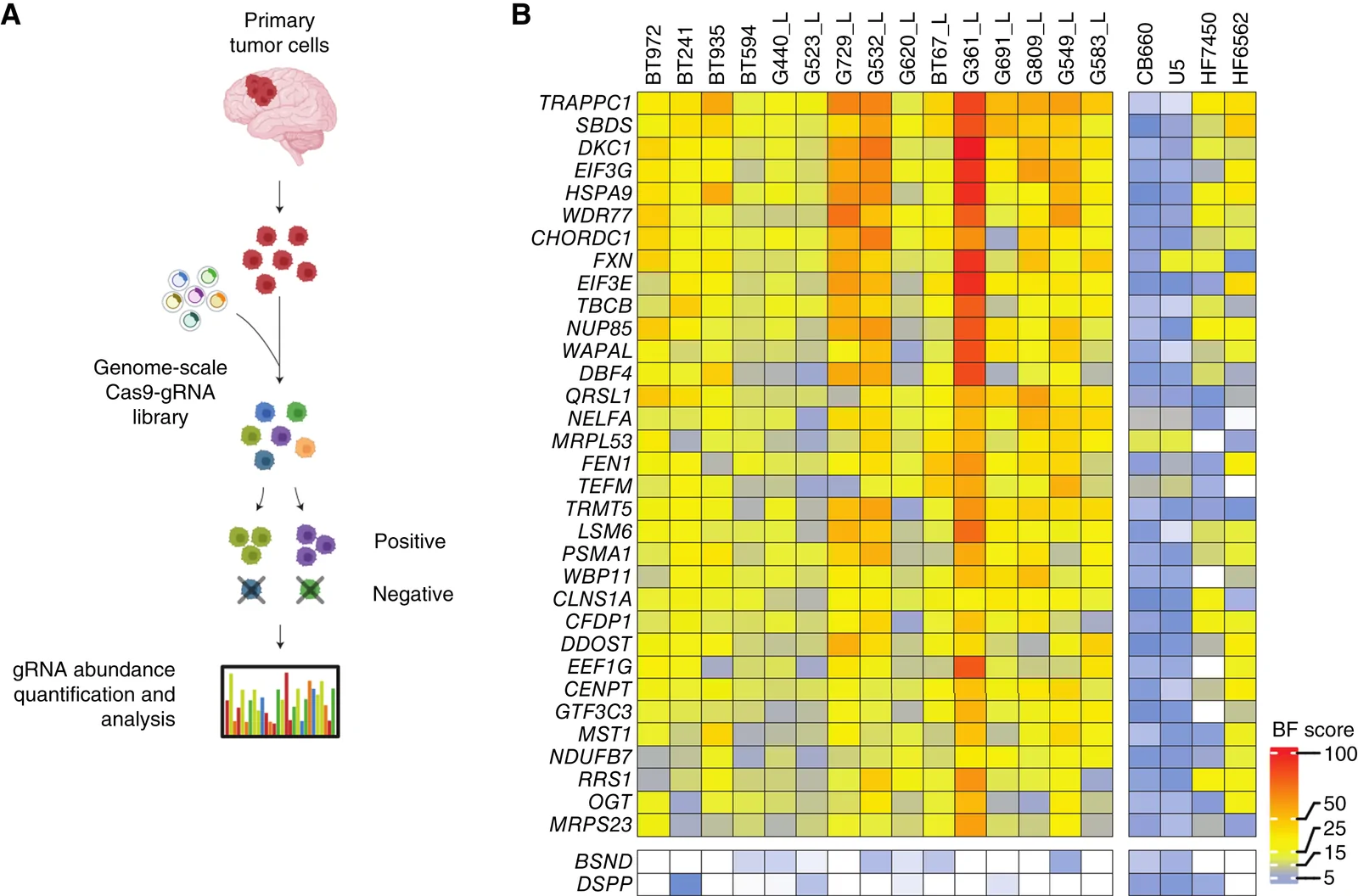
Genome-scale identification of GBM-enriched genetic dependencies. **A,** Schematic of genome-scale CRISPR-Cas9 knockout screening used to identify genetic dependencies in patient-derived GBM and NSC models. Cells were infected with a genome-wide CRISPR-Cas9 gene knockout library and cultured over 15 to 18 doublings. gRNA fold change within cell populations at the beginning and end of each screen was used to determine gene essentiality. **B,** BF scores for GBM-enriched genetic dependencies across 15 patient-derived GBM models and four NSC models.

To identify genetic drivers of GSC survival in a cancer-specific context, we conducted pooled, genome-wide CRISPR-Cas9 loss-of-function screening (Fig. 1A; Supplementary Fig. S1A and S1B) in a patient-derived GSC model (BT935) and compiled data from an additional 14 GSC models and four NSC models screened in our lab and by others (10–13). Beginning with genes essential across all GSC lines (BF ≥5, FDR < 0.05), those belonging to core essential gene sets (9, 14) and essential in a majority of NSC models were excluded, resulting in 33 genes of interest (Fig. 1B; Supplementary Table S2). The relative lack of GBM-enriched genetic dependencies highlights the large overlap of functional drivers between NSCs and GSCs and the significant heterogeneity between GBM models. The resulting GBM-enriched genetic dependencies included genes involved in DNA replication and repair (*FEN1* and *WAPAL*), RNA splicing (*LSM6* and *WBP11*), nuclear pore complex transport (*NUP85*), protein translation and ribosomal function (*EIF3G*, *EEF1G*, *EIF3E*, *NELFA*, *RRS1*, *MRPL53*, and *MRPS23*), and proteasomal degradation (*PSMA1*).

### FEN1 drives GSC proliferation and therapy resistance

We next investigated how these genetic dependencies contribute to therapy resistance. Essential genes elude conventional screens for therapy-sensitizing targets, as their loss impairs proliferation regardless of treatment. Consequently, their role in therapy resistance remains largely unexplored. To address this, we integrated the previous untreated screening data with chemoradiotherapy genetic screening, ranking GBM-enriched dependencies based on their differential effects between treated and untreated conditions. This approach enabled the identification of essential genes contributing to GSC therapy resistance.

**Figure 2.**
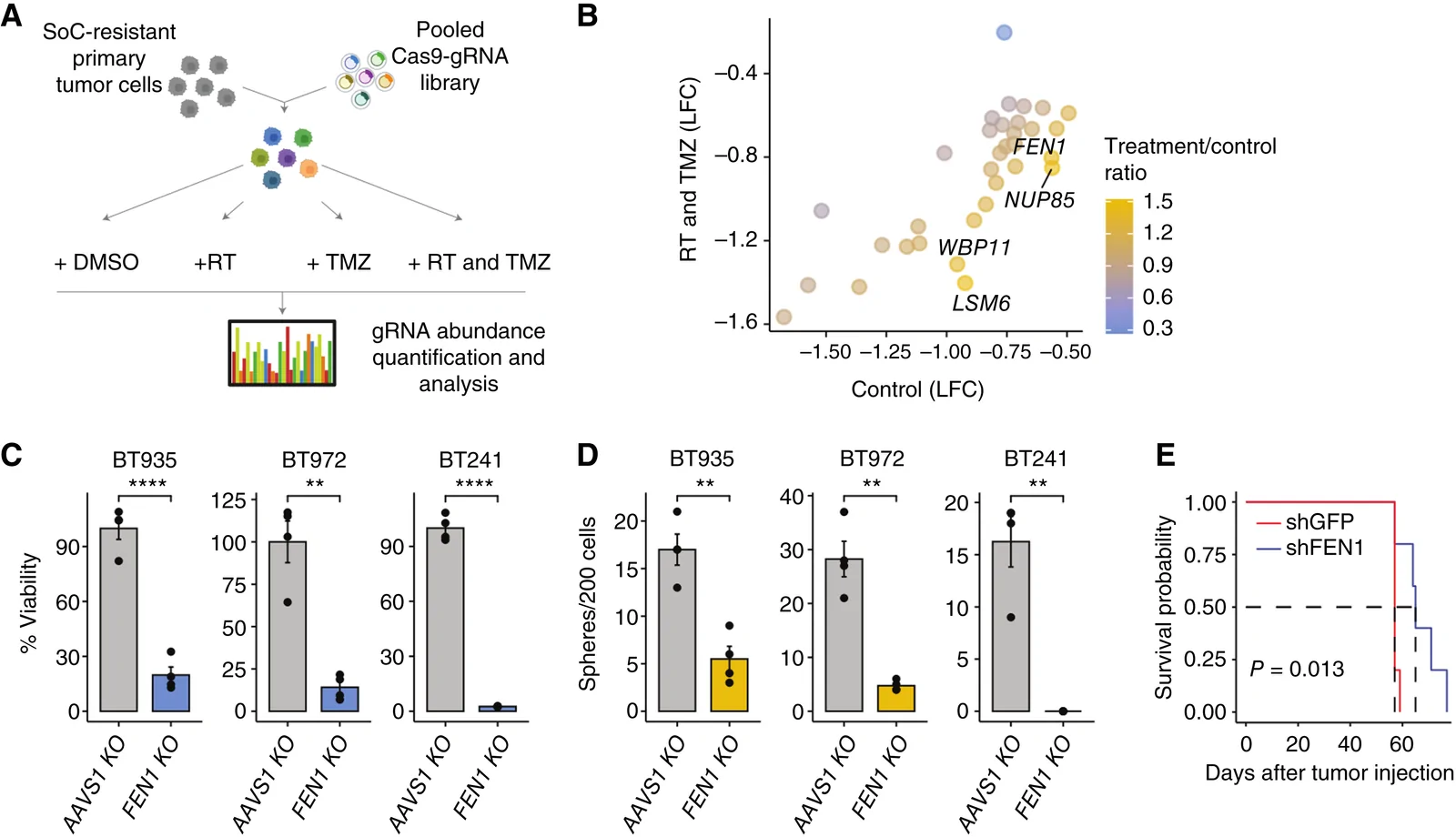
Identification of FEN1 as a driver of GSC proliferation and therapy resistance. **A,** Schematic of the chemoradiogenetic screen used to identify genetic dependencies of therapy resistance in the GBM model BT935. Cells were infected with a genome-wide CRISPR-Cas9 gene knockout (KO) library and exposed to DMSO, sublethal RT, sublethal TMZ, or combination treatment (RT and TMZ). gRNA LFC within cell populations at the beginning and end of each screen was used to determine gene essentiality. **B,** Comparison of gene-level LFC between DMSO and combination treatment arms in the GBM model BT935 for the 33 previously identified GBM-enriched genetic dependencies. Node color corresponds to the differential fitness ratio (LFC SoC/LFC DMSO), and genes with the highest enrichment in the therapy-treated arm are highlighted. **C** and **D,** Evaluation of *FEN1* KO on (**C**) proliferation and (**D**) sphere formation of GSCs, compared with a gRNA targeting *AAVS1* (control). Data are represented as mean ± SE; *n* = 4 experimental replicates; *P* values from unpaired *t* test (**, *P* < 0.01; ****, *P* < 0.0001). **E,** Kaplan–Meier survival analysis of immunocompromised mice engrafted with GBM tumor cells (BT972) with *FEN1* or *GFP* (control) knockdown (*n* = 5 per knockdown). *P* value was calculated using log-rank (Mantel–Cox) test.

Using the patient-derived GSC model BT935, we performed a genome-wide CRISPR-Cas9 loss-of-function screen under four conditions: vehicle control (DMSO), sublethal RT, sublethal TMZ, or combination therapy (Fig. 2A). Notably, fewer than 25% of conditional genetic interactions were shared across treatment arms, underscoring the specificity of resistance mechanisms and heterogeneity among drivers of genotoxic therapy resistance. To determine correlation across models, we compared our results with published data from seven additional primary GSC models treated with sublethal doses of TMZ (10, 12). Using the drugZ algorithm (15), we identified genetic interactions that significantly altered sensitivity to TMZ in primary GSC models and found little overlap among all eight cell lines, with or without filtering for genes that were significant hits in at least one screen (FDR < 0.05; Supplementary Fig. S2A). Given the uniqueness of genetic interaction profiles, we performed functional enrichment analysis (35) on internal primary GSC models (BT935 and BT594) to determine if changes in gene fitness showed similar trends at the pathway level. Enrichment analysis categorized treatment- and model-specific genetic interactions into distinct gene sets, with a single instance of overlap between models (Supplementary Fig. S2B; Supplementary Table S3; adjusted *P* < 0.05). The response to combination treatment in both primary GSC models highlights genetic interactions involved in DNA repair (BT594: *MSH2*, *PPP5C*, and *TP53*; BT935: *POLR2L*, *FANCA*, *FANCD2*, *FANCF*, *FANCM*, *and SLX4*), supported by known roles of MMR deficiency (36, 37) and a reliance on the Fanconi anemia (FA) pathway (12) in acquired chemotherapy resistance. To identify genes affecting fitness upon therapy exposure, we compared normalized gRNA read counts between SoC-treated and untreated arms using differential fitness ratios (LFC SoC/LFC DMSO; Fig. 2B; Supplementary Table S4). The top candidates were relatively unexplored in GBM and included genes involved in several processes traditionally associated with resistance to genotoxic therapies, such as DNA repair (MMR, HR), nuclear transport (nuclear pore complex), and RNA splicing, including the gene *FEN1*.

A key player in DNA replication and repair, FEN1 cleaves the 5′ ends of Okazaki fragments and processes DNA damage by cleaving 5′ overhanging nucleotides. It has also been implicated in apoptosis, telomere maintenance, and other important cellular functions, highlighting its role in genomic maintenance and stabilization (38, 39). Perturbation of *FEN*1 has been shown to sensitize tumor cells to DNA-methylating agents across multiple cancers, positioning it as a therapeutically relevant target (40–42). Although complete knockout of *FEN1* is embryonically lethal, heterozygous loss does not show severe effects, indicating a potential therapeutic window (43). Notably, FEN1 is highly expressed in proliferative tissues and cancers, correlating with advanced tumor grade and aggressiveness (42, 44–48). Given its role in homologous recombination and base excision repair, critical pathways for repairing TMZ-induced DNA damage, FEN1 emerges as a promising therapeutic target for overcoming resistance (49, 50).

Supporting our genomic observations and validating the effect of *FEN1* perturbation on the stem-like properties of GSCs, CRISPR-Cas9 knockout of *FEN1* significantly reduced proliferation and secondary sphere formation (measures of cell stemness) across several patient-derived GBM models (Fig. 2C and D; *P* < 0.05, unpaired *t* test, Supplementary Fig. S3A). Furthermore, although complete knockout is not feasible *in vivo* due to FEN1 essentiality, genetic knockdown of *FEN1* significantly extended the median survival of a patient-derived xenograft model of a highly aggressive, recurrent GBM (Fig. 2E; *P* < 0.05, Supplementary Fig. S3B).

### FEN1 dependency extends to TMZ resistance

**Figure 3.**
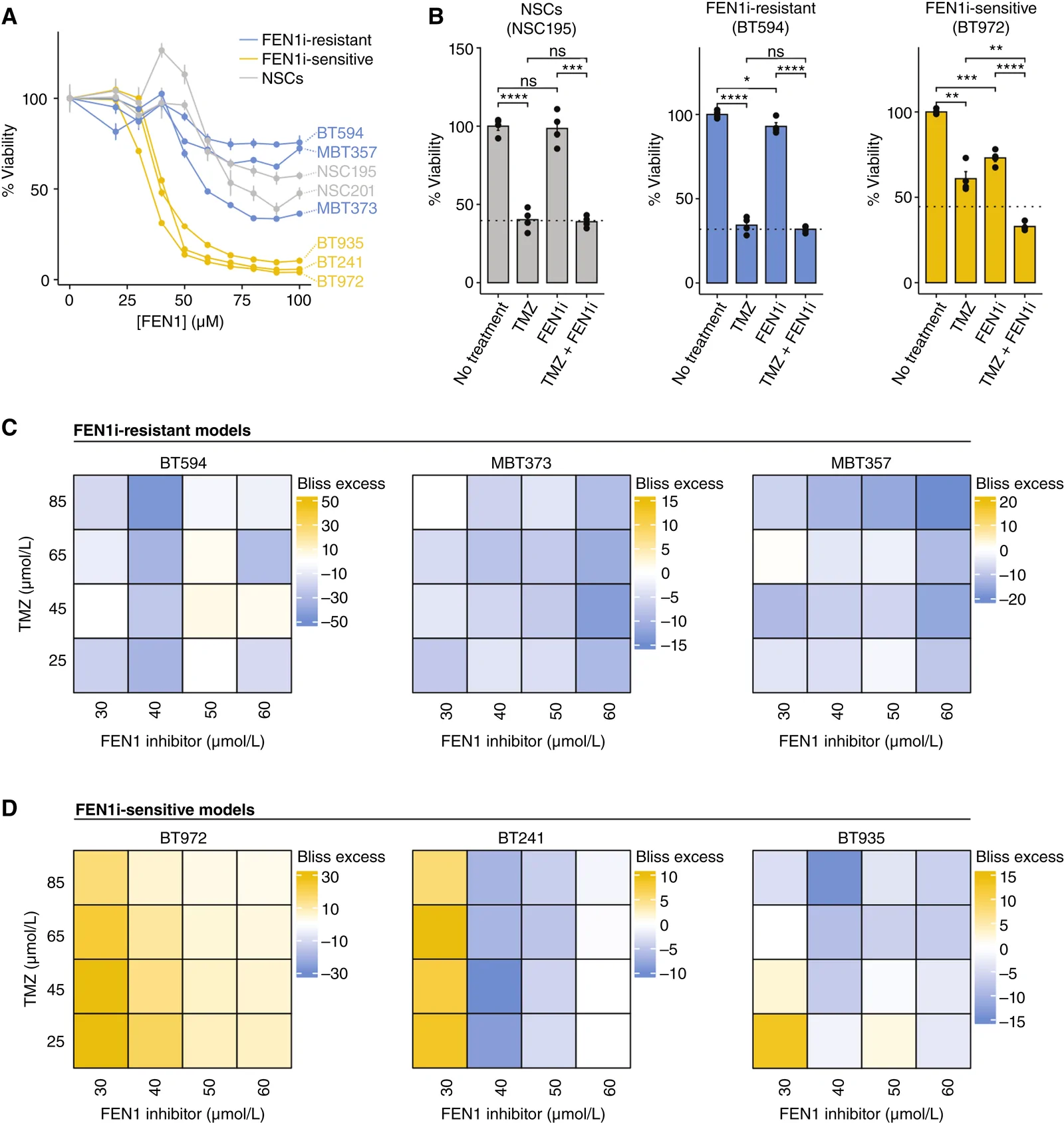
Sensitivity to FEN1 inhibition extends to TMZ sensitization. **A,** Cell viability assays of GSC and NSC lines exposed to increasing concentrations of FEN1 inhibitor over 7 days. Data are represented as mean ± SE; *n* = 4 experimental replicates. **B,** Cell viability assays of NSC, FEN1i-resistant, and FEN1i-sensitive GSC lines exposed to DMSO (control), 25 μmol/L TMZ, 30 μmol/L FEN1i, or combination (TMZ and FEN1i). Dashed lines represent expected viability under combination treatment. Data are represented as mean ± SE; *n* = 4 experimental replicates. **C** and **D,** Synergy assays between TMZ and FEN1 inhibitor in (**C**) FEN1i-resistant and (**D**) FEN1i-sensitive GBM models. Colors correspond to Bliss excess synergy scores, wherein more positive values indicate stronger synergy; *n* = 4 experimental replicates. ns, not statistically significant. *, *P* < 0.05; **, *P* < 0.01; ***, *P* < 0.001; ****, *P* < 0.0001.

To explore the therapeutic window of FEN1 perturbation, we acquired a small-molecule inhibitor of FEN1 [FEN1-in-4, compound 2 (51)]. FEN1 inhibition stratified six patient-derived GBM models and two healthy NSC models into two distinct phenotypes (Fig. 3A). Three GBM models showed sensitivity to FEN1 inhibition (herein called “FEN1i-sensitive”; BT972, BT241, and BT935), whereas three GBM models and the two NSC models showed resistance (herein called “FEN1i-resistant”; BT594, MBT357, MBT373, NSC195, and NSC201). Notably, the GBM models sensitive to FEN1 inhibition seemed to be the more aggressive models in our experience, including the two recurrent GBM models assayed.

To investigate canonical TMZ resistance markers across these models, *MGMT* promoter methylation status and MMR pathway features were assessed using methylation-specific PCR and RNA-seq, respectively. *MGMT* promoter methylation was seen in all cell lines sensitive to FEN1 inhibition (BT935, BT972, and BT241), as well as in one resistant line (BT594), but not in the remaining resistant lines (MBT357 and MBT373), as shown in Supplementary Fig. S4A. Expression of core MMR genes (*MSH2*, *MSH6*, *MLH1*, and *PMS2*) was detected in all cell lines, with no evidence of complete transcriptional loss. RNA-seq variant analysis identified a single truncating frameshift mutation in *MSH6* (T895*) in BT241, predicted to result in loss of function. No truncating or canonical splice-site mutations were detected in *MSH2*, *MLH1*, or *PMS2* in other cell lines; additional missense variants were classified as variants of uncertain significance. As matched DNA sequencing and microsatellite instability testing were not performed, MMR status was not assigned in a binary manner and is reported descriptively. Overall, canonical TMZ resistance markers did not clearly segregate with sensitivity to FEN1 inhibition across this panel.

To validate whether FEN1 contributes to GSC therapy resistance, we focused on TMZ over RT, given the greater reliance on FEN1 in the TMZ-treated arm of our screen compared with the RT-treated arm. Additionally, there was no correlation between sensitivity to FEN1 inhibition and TMZ, indicating that the two agents are suitable for combination with no cross-resistance (Supplementary Fig. S4B and S4C). Thus, we explored the effects of FEN1 inhibition on TMZ-induced cell death in both FEN1i-sensitive and FEN1i-resistant GSCs. We began by using a patient-matched primary-recurrent pair of GBM models, in which the primary GBM (BT594) was FEN1i-resistant, whereas the more aggressive, treatment-resistant recurrent GBM (BT972) was FEN1i-sensitive. Consistent with its prior exposure to SoC chemoradiotherapy, the recurrent GSCs were highly resistant to a therapeutically relevant concentration of TMZ compared with the primary counterpart and healthy NSCs (Fig. 3B; refs. 52–54). In contrast, this model was more sensitive to FEN1 inhibition, suggesting that FEN1 dependency may have been acquired upon recurrence, and it was the only model that FEN1 inhibition sensitized to TMZ (*P* < 0.05, unpaired *t* test). This prompted us to investigate this synergy across all six GBM models previously assayed. Although no synergy with TMZ was present in the three FEN1i-resistant GBM models (Fig. 3C), there was strong sensitization to TMZ-induced killing at low concentrations of FEN1 inhibitor across all three FEN1i-sensitive GBM models, indicated by synergistic cell death with high Bliss excess (Fig. 3D). These results demonstrate that sensitivity to FEN1 inhibition extends to therapy sensitization, selectively in a subset of GSCs, and position FEN1 as a cancer-selective, therapy-sensitizing target in highly aggressive GBMs.

### FEN1 inhibition mediates TMZ sensitization through increased DNA damage

**Figure 4.**
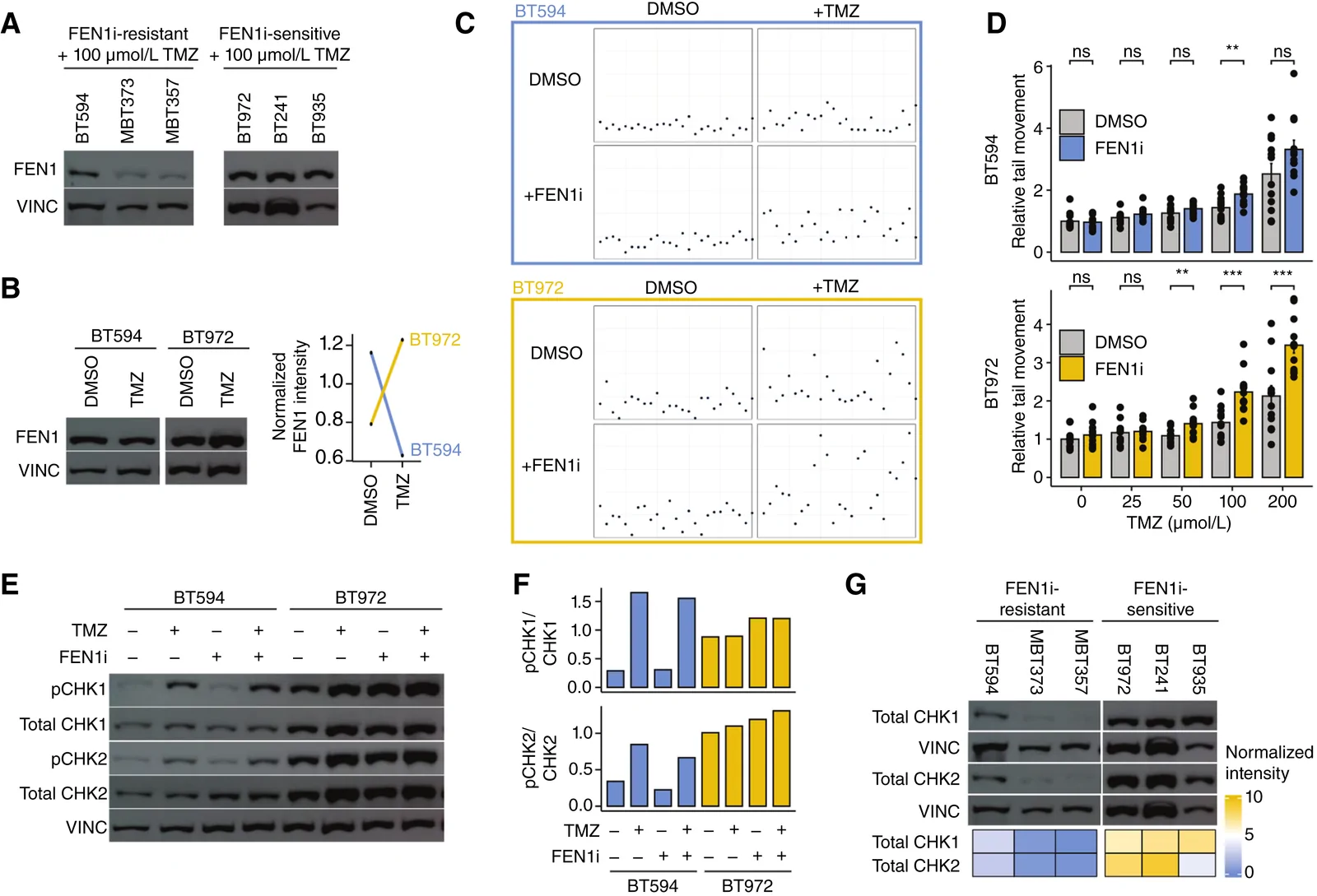
FEN1 inhibition enhances TMZ-induced DNA damage. **A,** Immunoblotting of FEN1i-resistant and FEN1i-sensitive GSCs for FEN1 protein after treatment with 100 μmol/L TMZ (*n* = 3 biological replicates per group). **B,** Immunoblotting of patient-matched primary (FEN1i-resistant; BT594) and recurrent (FEN1i-sensitive; BT972) GSCs for FEN1 protein after treatment with DMSO (control) or 100 μmol/L TMZ. FEN1 signal intensity normalized to vinculin control is shown for the representative immunoblot. **C,** Scatterplots indicating representative individual cellular comet tail moment values of FEN1i-resistant (BT594) and FEN1i-sensitive (BT972) GSCs from each corresponding cell/treatment type: DMSO alone (control), TMZ (200 μmol/L), FEN1i (30 μmol/L), or combination (TMZ–FEN1i, 200 μmol/L/30 μmol/L). **D,** Quantification of comet assays. Relative tail movement of FEN1i-resistant and FEN1i-sensitive GSCs is shown with increasing concentrations of TMZ ± 30 μmol/L FEN1 inhibitor for 48 hours. Data are represented as mean ± SE; *n* = 12 experimental replicates. **E,** Immunoblotting of FEN1i-resistant (BT594) and FEN1i-sensitive (BT972) GSCs for phosphorylated CHK1, total CHK1, phosphorylated CHK2, and total CHK2 after a 3-day treatment with DMSO (control), TMZ (45 μmol/L), FEN1i (30 μmol/L), or combination (TMZ and FEN1i). **F,** Quantification of immunoblotting in **E**. The proportion of phosphorylated (active) CHK1 and CHK2 is shown, with signal intensities normalized to vinculin control. Quantification is provided from the representative immunoblot shown to facilitate comparison across the indicated treatment conditions; independent replicate immunoblot experiments were not performed for this analysis. **G,** Immunoblotting of FEN1i-resistant and FEN1i-sensitive GSCs (*n* = 3 biological replicates per grouping) for total CHK1 and total CHK2 protein after treatment with TMZ (100 μmol/L). Heatmap color corresponds to normalized FEN1 intensity. All samples of each individual experiment were run on the same immunoblot under identical experimental conditions, with immunoblots cropped for presentation. Quantification of immunoblots is presented for visualization of relative signal intensity only and should not be interpreted as statistical comparisons across experiments. ns, not statistically significant. **, *P* < 0.01; ***, *P* < 0.001.

We next explored this sensitization to TMZ at the level of DNA damage and DNA damage repair. Upon exposure to a high dose (100 μmol/L) of TMZ, FEN1 protein expression was significantly higher in FEN1i-sensitive GSCs (Fig. 4A; Supplementary Fig. S5A). Comparing the patient-matched primary-recurrent GBM models, TMZ treatment increased FEN1 protein expression only in the recurrent GSCs, suggesting a greater reliance on FEN1-mediated DNA repair processes in response to TMZ (Fig. 4B). To determine how FEN1 inhibition enhances TMZ activity, we proceeded to quantify DNA double-strand breaks (DSB) and assay the expression of several DNA damage response proteins involved in DSB repair after exposure to TMZ, FEN1 inhibition, or a combination treatment. Using a comet assay to quantify DSBs, exposure to 30 μmol/L FEN1 inhibitor did not induce DSBs in either GBM model (Fig. 4C and D; Supplementary Fig. S5B). However, this concentration of the FEN1 inhibitor did enhance DSBs induced by increasing concentrations of TMZ, markedly more so in the recurrent GBM, confirming that inhibition of FEN1 sensitizes GSCs to TMZ by enhancing DNA damage. Looking at the DNA damage repair proteins, changes in phosphorylated (active) Chk1, Chk2, and ATM mimicked our functional findings (Fig. 4E and F; Supplementary Fig. S5C and S5D). Although the FEN1i-resistant GSCs showed a significant DNA repair response to TMZ and no response to FEN1 inhibition, the FEN1i-sensitive GSCs showed a greater response to FEN1 inhibition, which carried over to the combination treatment arm. Interestingly, in contrast to a previously identified synthetic lethality between FEN1 and DNA protein kinase (DNA-PK) in glioma, changes in phosphorylated DNA-PK protein levels did not discriminate between the primary and recurrent GBM (Supplementary Fig. S5D), indicating that the effects of FEN1 inhibition are independent of nonhomologous end joining. Confirming these findings across multiple GBM models, Chk1 and Chk2 phosphorylation was significantly higher in FEN1i-sensitive GSCs after treatment with 100 μmol/L TMZ compared with FEN1i-resistant GSCs (Fig. 4G). These data support that FEN1 inhibition targets this subset of GBMs and enhances TMZ efficacy by increasing TMZ-induced DSBs.

### FEN1 maintains GSC stemness and proliferation

**Figure 5.**
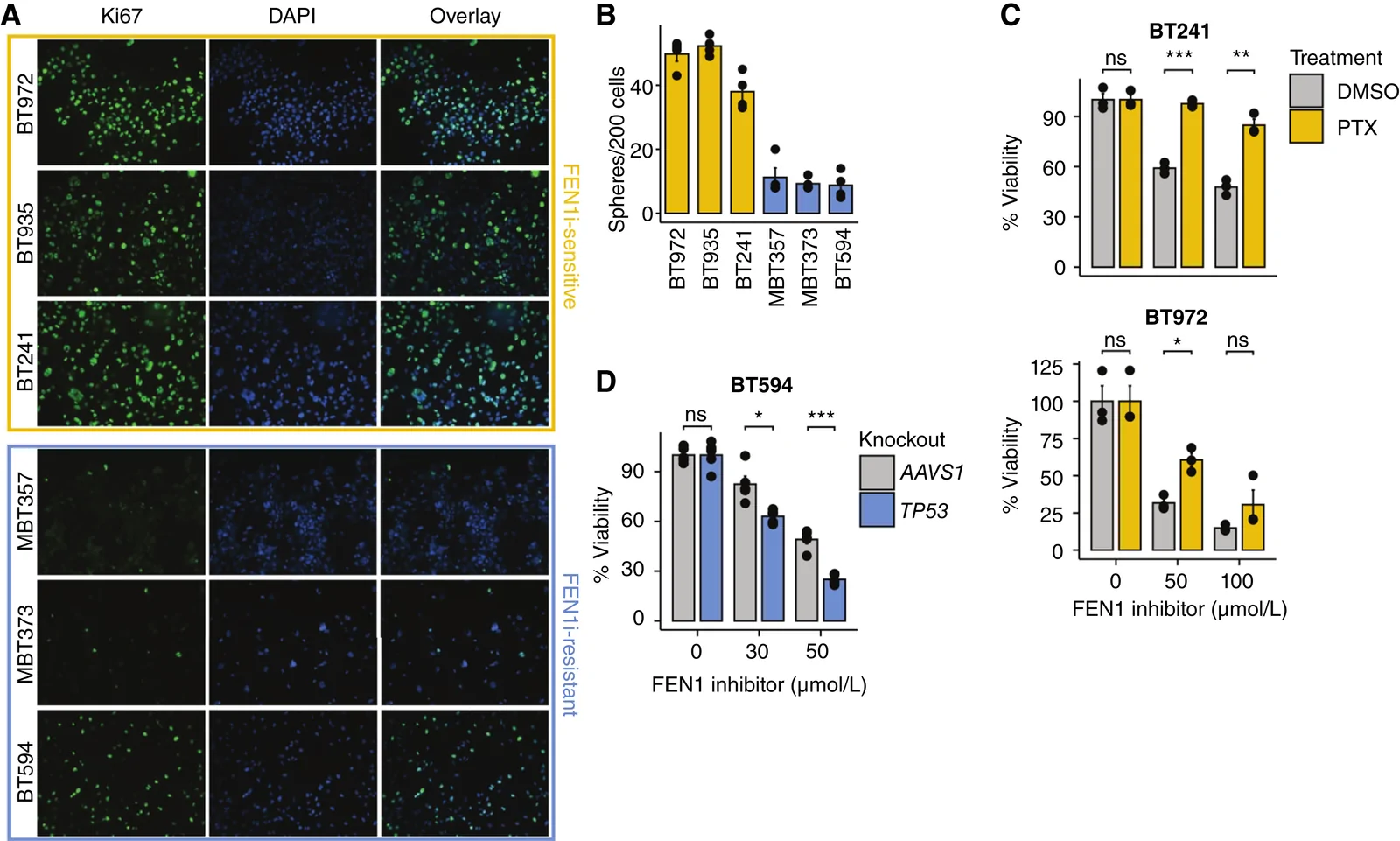
Sensitivity to FEN1 inhibition is associated with tumor cell stemness and proliferation. **A,** Immunofluorescence staining for Ki67 and DAPI in FEN1i-sensitive (BT972, BT935, BT241) and FEN1i-resistant (MBT357, MBT373, BT594) GSCs. **B,** Secondary sphere formation assay in FEN1i-sensitive and FEN1i-resistant GSCs. Data are represented as mean ± SE; *n* = 4 experimental replicates. **C,** Cell viability assays of FEN1i-sensitive GSC lines cultured with or without PTX (cell-cycle stalling agent) for 6 days, exposed to increasing concentrations of FEN1i. Data are represented as mean ± SE; *n* = 3 experimental replicates. **D,** Cell viability assay of FEN1i-resistant GSCs with *TP53* or *AAVS1* (control) knockout, exposed to increasing concentrations of FEN1i. Data are represented as mean ± SE; *n* = 5 experimental replicates. ns, not statistically significant. *, *P* < 0.05; **, *P* < 0.01; ***, *P* < 0.001.

We next sought to characterize the determinants of FEN1 dependency. Comparing stemness properties, FEN1i-resistant GSCs had higher immunofluorescent staining of the proliferation marker Ki67 (Fig. 5A; Supplementary Fig. S6) and exhibited a higher sphere-forming capacity (Fig. 5B). To more directly establish a relationship between proliferation and reliance on FEN1, we treated highly proliferative FEN1i-sensitive GSCs with PTX, an inhibitor of mitosis and cell division, which stalled cell proliferation while maintaining viability. PTX treatment resulted in significant resistance to FEN1 inhibition (Fig. 5C), indicating the importance of FEN1 to proliferative cells. To further show this relationship, we did the opposite and hyper-proliferated slow-dividing FEN1i-resistant GSCs using a *TP53* knockout, a common mutation found at GBM recurrence. Doing so sensitized the FEN1i-resistant GSCs to FEN1 inhibition (Fig. 5D), further supporting the importance of FEN1 function in proliferative cells.

**Figure 6.**
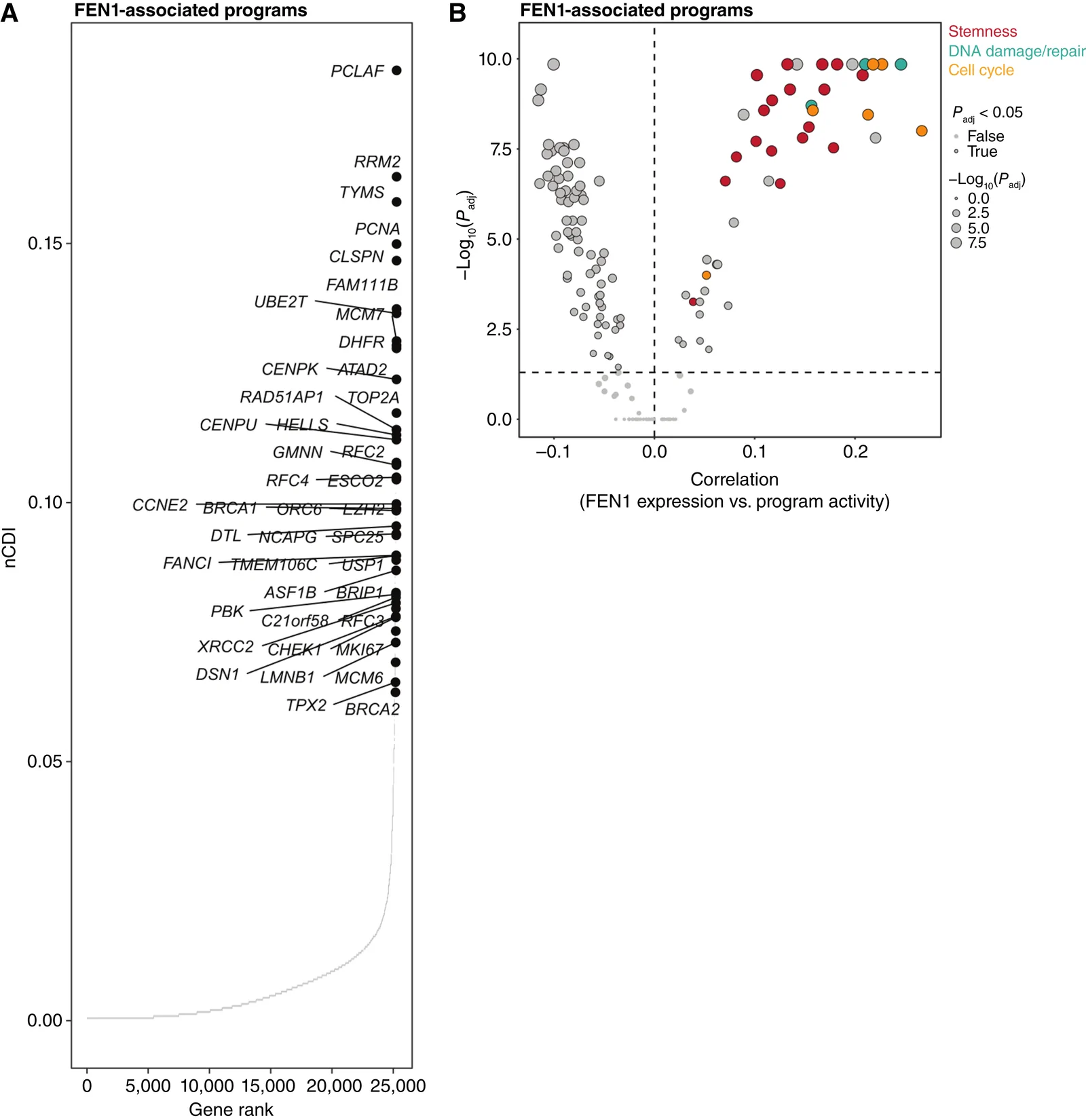
Single-cell analysis reveals that *FEN1* expression correlates with key genes and gene programs involved in GBM proliferation, stemness, and DNA damage repair. **A,** Codependency analysis of *FEN1* expression, with coexpression represented by nCDI. Key genes are highlighted and labeled, indicating strong coexpression with *FEN1*. **B,** Gene program analysis showing correlation between *FEN1* expression and program activity. Dot size corresponds to −log_10_ (adjusted *P* value), with larger dots indicating more statistically significant results. Dots are color-coded according to gene program category, and key programs are highlighted.

To characterize the association of FEN1 expression with defined cellular programs within heterogeneous GBM populations, we analyzed scRNA-seq data from patient-derived GBM cell lines from the Abdelfattah and colleagues (19) and Wang and colleagues (20) datasets. At the gene level, *FEN1* expression correlated strongly with key regulators of cell-cycle progression, stemness, and DNA damage repair (Fig. 6A; Supplementary Table S5). Consistent with our prior immunofluorescence findings, one of the strongest codependencies was *MKI67*, a widely used marker of proliferation. Other proliferation-associated genes included *PCNA*, a master regulator of DNA replication, and *CCNE2*, which also promotes rapid cell-cycle progression. Additionally, multiple genes involved in mitosis, chromosomal segregation, and spindle assembly, including *TPX2*, *SPC25*, *DSN1*, *DTL*, and *NCAPG*, were highly coexpressed with *FEN1*. From a stemness perspective, *FEN1* correlated with key GBM stemness-associated genes, including *EZH2*, *ATAD2*, and *HELLS*, which epigenetically regulate cancer cell plasticity and self-renewal. Additionally, *ASF1B and PBK*, which contribute to stem-like properties and tumor progression, were highly coexpressed. *FEN1* also showed strong associations with genes involved in DNA damage repair pathways, particularly homologous recombination, including *BRCA1*, *BRCA2*, *RAD51AP1*, and *XRCC2.* Genes within the FA pathway, such as *BRIP1*, *FANCI*, *UBE2T*, and *USP1*, were also highly coexpressed, further supporting a role for *FEN1* in maintaining genomic stability. Finally, *CHEK1*, a master checkpoint kinase essential for the DNA damage response, was among the top coexpressed genes.

At the gene program level, *FEN1* expression was again significantly enriched in programs related to cell cycle/proliferation, stemness, and DNA damage repair, further reinforcing its role in aggressive GBM biology (Fig. 6B; Supplementary Table S6). Notably, proliferation and cell-cycle programs, including MP1_CellCycle_G2M (23), MP2_CellCycle_G1S (23), MP3_CellCycle_HMG (23), CellCycle_Cancer (26), and HALLMARK_G2M_CHECKPOINT (55), were highly correlated with *FEN1* expression. Stemness programs, such as StemSig_Zhang2022 (28), Nanog_Benporath (30), Sox2_Benporath (30), Human_Epithelial_ASC_Smith (56), and hESC_Bhattacharya (57), were also enriched, highlighting *FEN1*’s association with GSC identity. Finally, DNA damage repair pathways, including HALLMARK_DNA_REPAIR (55), DNArepair_CancerSEA (26), and DNAdamage_CancerSEA (26), were highly expressed in GBM cells expressing *FEN1*, consistent with its role in genomic maintenance.

These findings suggest that *FEN1* is a central regulator of proliferation, stemness, and DNA damage repair in GBM, making it a promising therapeutic target, particularly in combination with DNA-damaging agents such as TMZ.

## Discussion

Despite decades of progress in cancer biology and therapeutics, GBM remains one of the most treatment-refractory and deadly cancers, underscoring the urgent need for innovative therapeutic strategies. Functional genomics has begun to shed light on therapy resistance in GBM. MacLeod and colleagues (12) previously implicated FA and homologous recombination pathways (including genes such as *FANCA*, *C19orf40*, *MCM8*, and *MCM9*) as contributors to TMZ resistance in primary GSCs. Similarly, Rocha and colleagues (37) highlighted resistance pathways involving Sonic Hedgehog signaling, circadian rhythm genes, the NRF2 antioxidant response, and Wnt/β-catenin signaling, identifying genes such as *CLCA2*, *PTCH2*, *FZD6*, and *CTNNB1*, which promote cell survival when overexpressed, suggesting they could be targeted to improve TMZ efficacy. Despite these advances, the functional genetic regulators underpinning GSC resistance remain poorly understood, and actionable therapeutic targets are scarce. In this study, we conducted the first functional genetic screening in patient-derived GBM models to investigate essential gene modulators of SoC, uncovering FEN1 as a critical driver of survival and TMZ resistance in an aggressive subset of GBMs. Our findings position FEN1 as a cancer-selective therapeutic target with the potential to enhance the efficacy of SoC in aggressive, treatment-refractory GBM.

Our chemoradiogenetic screens in the BT935 primary GSC model revealed that GSCs display condition-specific dependency profiles, underscoring the complexity of therapy resistance mechanisms. We propose that resistance may arise from broad, essential genetic programs responsible for tumorigenesis, growth, and survival, which have thus far eluded genomic efforts. The preferential sensitization of FEN1 inhibitor-sensitive GSCs to TMZ by FEN1 inhibition supports a model in which genes essential for tumorigenic properties further shield tumor cells from genotoxic therapies. Notably, although FEN1 was identified as a shared genetic dependency across all GSC models, only a subset demonstrated sensitivity to pharmacologic FEN1 inhibition. This distinction highlights a disconnect between genetic essentiality and inhibitor response, suggesting that context-dependent factors or threshold effects influence whether FEN1 inhibition translates into a measurable therapeutic vulnerability. To further contextualize these findings at the cellular state level, we leveraged scRNA-seq data from patient-derived GBM models to assess whether *FEN1* expression is associated with defined cellular programs within heterogeneous tumor populations. These analyses suggest that FEN1 expression is enriched in proliferative and stem-like cellular states, consistent with its proposed role in sustaining tumorigenic capacity and therapy resistance. These findings highlight the potential of targeting fundamental tumorigenic dependencies to overcome resistance and should prompt further exploration into the involvement of essential genes in these processes.

Targeting cancer stem cell properties in GBM has long been challenging due to the homology with healthy NSCs. Additionally, the clinical outcomes of NSC toxicity are uncertain, and although NSCs remain largely quiescent after development, ample evidence supports their roles in aging, plasticity, disease, and regeneration of the nervous system. Despite these challenges, several preclinical and clinical strategies have aimed to target stem-like properties in GBM, including BMI1 and EZH2 inhibitors and CD133 chimeric antigen receptor T cells (58, 59). Our study contributes to this effort by advancing the understanding of GSC-specific dependencies, particularly those related to DNA damage response pathways. These pathways, which maintain mutational tolerance and genomic stability, are critical for maintaining the plasticity and stemness of cancer stem cells. Reliance on these proteins presents a targetable dependency enriched by exposure to genotoxic therapies, and FEN1 emerges as a key dependency within this framework, representing a novel and actionable vulnerability in GSCs.

In addition to deciphering cancer-specific stem cell biology, the field is further complicated by the temporal evolution GBM undergoes throughout the course of therapy. It has been well established that recurrent GBM is markedly different from the primary disease, being more aggressive and more resistant to a broad range of therapeutics, and a lack of scientific discovery in recurrent GBM models likely contributes to the failures seen across clinical trials (60, 61). Thus, therapeutic strategies that maintain or enhance efficacy in the highly proliferative, therapy-resistant phenotype characteristic of recurrent GBM are crucial. Additionally, upon acquiring this resistant phenotype, patients with recurrent GBM often refuse secondary TMZ treatment due to a lack of efficacy. Identifying therapeutic strategies that are effective against recurrent GBM and further address this resistance to therapy may improve the efficacy of secondary TMZ and allow it to extend patient survival. However, we note that our analysis of primary–recurrent differences is based on a single patient-matched pair and therefore should be interpreted cautiously in the context of the substantial intertumoral heterogeneity observed in GBM.

Although our study provides compelling evidence for FEN1 as a novel therapeutic vulnerability in GSCs, we are limited by a lack of *in vivo* validation, which remains a critical next step in translating these findings toward clinical impact. Notably, the lack of blood-brain barrier (BBB)–penetrant FEN1 inhibitors poses a current limitation to such studies; however, this challenge also represents an exciting and necessary opportunity for future drug development. Given the urgent clinical need for novel, effective treatments in GBM, particularly for recurrent, treatment-resistant disease, the development of BBB-penetrant FEN1 inhibitors could enable preclinical *in vivo* studies and ultimately catalyze the translation of our findings into viable clinical strategies. As such, FEN1 represents both a promising therapeutic target and a foundation for future innovations in GBM drug discovery.

In summary, our study highlights FEN1 as a pivotal driver of GSC survival and therapy resistance, with dual roles in maintaining tumorigenic properties and mediating genotoxic therapy resistance. By targeting this dependency, we provide a foundation for novel therapeutic strategies aimed at disrupting critical vulnerabilities of stemness in GBM, particularly in its most aggressive and treatment-resistant forms.

## Supplementary Material

Supplementary Table 1 Antibodies used for immunoblotting

Supplementary Table 2 Bayes factors for CRISPR-Cas9 dropout screens, related to figure 1B

Supplementary Table 3 ClusterProfiler analysis of conditional GIs (drugZ FDR < 0.05) from all screens, related to supplementary figure 2B

Supplementary Table 4 Normalized gRNA read counts for chemoradiogenetic CRISPR-Cas9 screen in GBM model BT935, related to figure 2B

Supplementary Table 5 Genes associated with *FEN1* expression in scRNA-seq analysis, related to figure 6A

Supplementary Table 6 Programs associated with *FEN1* expression in scRNA-seq analysis, related to figure 6B

Supplementary Figure 1 Quality control analysis of CRISPR-Cas9 dropout and chemoradiogenetic screens in GBM model BT935, related to figure 1B and 2B. A) Gene-level LFC of CEGs and nonessential genes across treatment arms. All data presented from n = 3 biologically independent replicates. P values are from unpaired Student’s t tests. B) Precision-recall plots indicate high performance of screens across treatment arms. C) Number of essential genes (FDR < 0.05) identified in screens across treatment arms. D) Number of conditional genetic interactions identified in screens across treatment arms (FDR < 0.05).

Supplementary Figure 2 A) Pearson correlation of genetic interaction (drugZ) scores from all treated screens, presented with (right) and without (left) filtering for genes with false discovery rate (FDR) < 0.05 in at least one screen. B) ClusterProfiler analysis of cGIs (drugZ FDR < 0.05) from all screens. Reactome gene sets enriched at an adjusted P < 0.05 are shown in the dot plot, with dot size corresponding to gene ratio and dot color indicating adjusted P values. Positive and negative cGIs responsible for the only overlapping enriched pathway (DNA Repair) are listed for each model.

Supplementary Figure 3 A) Immunoblotting of FEN1 protein in GSCs after knockout of *FEN1* or *AAVS1* (control), related to figure 2C and 2D. B) Immunoblotting of FEN1 protein in GSCs after knockdown of *FEN1* or *GFP* (control), related to figure 2E.

Supplementary Figure 4 A) Methylation-specific PCR (MSP) gel showing *MGMT* promoter methylation status across GBM cell lines. Methylated (M) and unmethylated (U) primer sets were used following bisulfite conversion. Commercial methylated and unmethylated DNA controls and a no-template water control (H_2_O) were included as assay controls. B) Cell viability assays of GSC lines exposed to increasing concentrations of TMZ inhibitor over 7 days, related to figure 3. Data are represented as mean ± s.e.; n = 4 experimental replicates. C) Plot of TMZ and FEN1i IC50 values, related to figure 3.

Supplementary Figure 5 A) Normalized intensities of FEN1 protein from immunoblotting related to figure 4A. B) Representative images of comet assays of FEN1i-resistant (BT594) and sensitive (BT972) GSCs exposed to DMSO alone (control), TMZ (200 µM), FEN1i (30 µM) or combination (TMZ/FEN1i, 200 µM/ 30 µM). C) Normalized intensities of phospho-Chek1, total Chek1, phospho-Chek2 and total Chek2 protein from immunoblotting related to figure 4E and 4F. D) Immunoblotting for phospho-ATM, total ATM, phospho-DNAPK and total DNAPK protein normalized to vinculin control.

Supplementary Figure 6 Quantification of KI67 staining, as percent-positive for KI67, related to figure 5A.

## Data Availability

Processed data are included in the article and its supplementary materials or are available from the corresponding author upon reasonable request. Bulk RNA-seq data generated in this study have been deposited in the NCBI GEO database under accession number GSE347233. Previously published bulk RNA-seq data for the BT594 and BT972 cell lines are available under GEO accession number GSE240490. All unique biological resources are available from the corresponding author upon request.

## Code Availability

All custom codes used are available on GitHub (https://github.com/NMikolajewicz/Brakel-2025).
